# Lung ultrasound to evaluate the outcome and prognosis of transient tachypnea of the newborn

**DOI:** 10.3389/fped.2024.1536992

**Published:** 2025-01-17

**Authors:** Peng Jiang, Jing Wei, Meiying Han

**Affiliations:** ^1^ Department of Pediatrics, Liaocheng People’s Hospital, Liaocheng, Shandong, China; ^2^ Department of Ultrasound, Liaocheng People’s Hospital, Liaocheng, Shandong, China

**Keywords:** lungs, pulmonary, ultrasonography, newborn, transient tachypnea of the newborn

## Abstract

**Background/Objectives:**

Transient tachypnea of the newborn (TTN) is generally self-limiting. The increasing incidence of severe cases and lack of comprehensive studies on its clinical outcomes and influencing factors highlight the need for improved diagnostic and monitoring tools. This study aimed to assess the lung ultrasonographic features, recovery durations, and factors influencing TTN.

**Methods:**

Two hundred neonates with wet lungs were selected as the TTN group and divided into mild (132/200) and severe (68/200) groups. Two hundred neonates without pulmonary disease served as controls. The acute-phase lung ultrasonographic features of the two groups were compared. According to the clinical recovery duration, TTN group was divided into group A (<5 days, 191/200) and group B (≥5 days, 9/200). Univariate and multivariate logistic regression analyses were used to analyze the relationship between clinically relevant factors and the recovery time.

**Results:**

The time that was required for the 200 neonates to fully recover without symptoms was 2.3 ± 1.33 days. The average clinical recovery durations of neonates with mild illness, those who required non-invasive ventilation, and those who required invasive ventilation were 1.42 ± 0.50, 3.36 ± 0.51, and 6.00 ± 1.12 days, respectively. The differences between the groups were significant (*P* < 0.05). Type II respiratory failure, acute respiratory distress syndrome (ARDS), persistent pulmonary hypertension of the newborn (PPHN), and heart failure were important factors affecting the recovery time.

**Conclusion:**

The lung ultrasonographic signs differed based on the degree of wet lungs. Moreover, type II respiratory failure, ARDS, PPHN, and heart failure are important factors prolonging the course of TTN in neonates.

## 1 Introduction

Transient tachypnea of the newborn (TTN) is a self-limiting respiratory disease caused by the accumulation and delayed clearance of fluids in the lungs. It mainly manifests as dyspnea and is a common cause of early neonatal respiratory distress (1). In most cases, this disease is self-limiting, does not require intervention, and has a good prognosis. However, the number of severe wet lung cases is increasing annually, and clinical respiratory support is usually required (2–4).

In recent years, lung ultrasonography (LUS) has been used for the diagnosis and differential diagnosis of TTN and has high sensitivity and specificity (5, 6). However, few studies have reported the outcomes of TTN and its influencing factors (7). Therefore, this study aimed to investigate the LUS features associated with varying degrees of wet lungs, dynamically and continuously monitor the pulmonary changes in neonates with TTN using LUS and combine these findings with clinical indicators to assess the progression of clinical outcomes. This approach is intended to contribute to the improved scientific management of TTN.

## 2 Materials and methods

### 2.1 Participants

A retrospective study was conducted on 200 children with transient tachypnea of the newborn (TTN) who were admitted to the neonatal ward of Liaocheng People's Hospital from March 1, 2022 to March 31, 2024. Depending on whether the neonates required mechanical ventilation, they were classified as having a mild or severe condition (8). Neonates whose main clinical manifestation was tachypnea and those who only needed general oxygen therapy or did not need oxygen therapy without mechanical ventilation were classified as having a mild condition. Those with obvious dyspnea who required ventilator-assisted breathing [including continuous positive airway pressure (CPAP) and tracheal intubation-assisted breathing] were classified as having a severe condition. We divided 200 children with TTN into Group A (<5 days, 191/200) and Group B (≥5 days, 9/200).

The inclusion criteria (9) were as follows: (1) Meeting the diagnostic criteria for neonatal wet lungs: The diagnosis was made comprehensively by a clinician uninvolved in this study based on medical history, clinical manifestations, arterial blood gas analysis, chest radiography, and LUS examination. All 200 neonates were diagnosed with TTN using chest radiography and LUS immediately after admission. Chest radiography revealed decreased bilateral lung transparency, patchy exudative shadows, reticular thickening, alveolar and interstitial fluid accumulation, pulmonary congestion, emphysema, and interlobar and pleural effusion. Lung ultrasound showed signs of alveolar fluid accumulation, including alveolar-interstitial syndrome (AIS) or double lung points, an abnormal pleural line, disappearance of the A-line, and no lung consolidation. The clinical criteria for normal recovery included the resolution of tachypnea or dyspnea symptoms, normalization of arterial blood gas analysis for at least 12 h, and confirmation through LUS examination (i.e., the pleural line appeared smooth and clear; the A-line exhibited a parallel, “bamboo-like” strong echo; and there were either no B-lines or only a small number of B-lines in the lung field) (8, 10). (2) Admission within 2 h of birth.

The exclusion criteria were as follows: (1) gestational age <34 weeks (9 cases), (2) incomplete data owing to various reasons (3 cases), and 3) transfer to another hospital during the course of treatment (2 cases).

Two hundred neonates without pulmonary disease admitted during the same period were selected as the control group. This study complied with the relevant medical ethics regulations of the Declaration of Helsinki and was approved by the Medical Ethics Committee of Liaocheng People's Hospital. Informed consent was obtained from the neonates’ parents.

**Figure 1:**
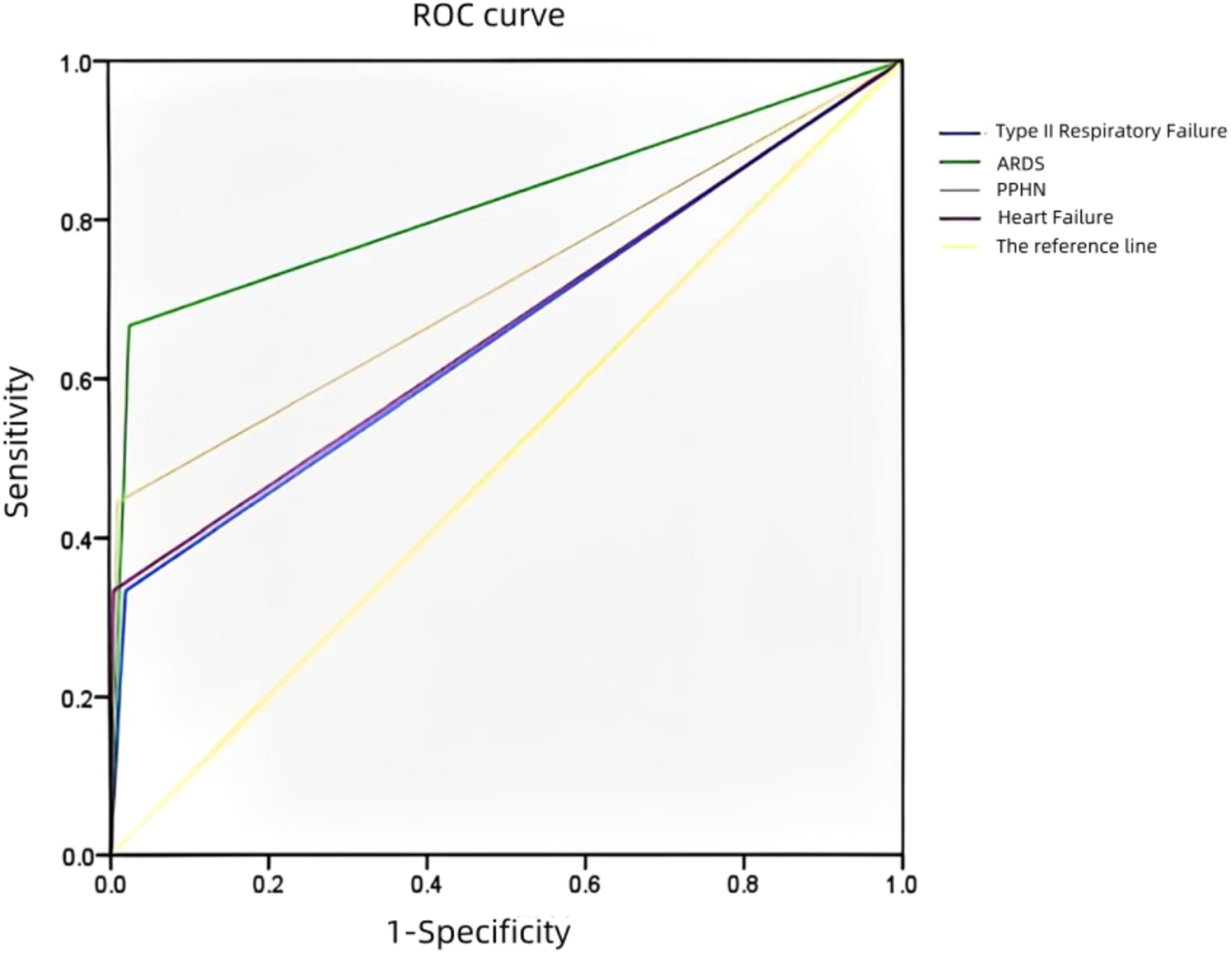
Flowchart of enrollment. TTN, transient tachypnea of the newborn.

Figure 1 illustrates the enrollment ﬂowchart.

### 2.2 Clinical data collection

The clinician designed a form and recorded relevant information, such as name, hospital number, sex, age at admission to the department, gestational age, birth weight, prenatal ultrasound information, delivery mode, type of respiratory support, type of complications, and respiratory rate, according to the relevant literature and clinical work experience during the first ultrasound examination.

### 2.3 Examination methods

All neonates were examined using bedside ultrasonography by the same group of physicians trained in neonatal lung ultrasonography. The first examination was performed within 6 h after admission. Re-examinations were performed every 24 h, starting at 12 and 24 h after admission, until the LUS findings returned to normal. The lung ultrasound examination was conducted following the 2019 International Guidelines for Neonatal Lung Ultrasound Examination (11), using a MylabTwice ultrasound system (Esaote, Italy) equipped with a high-frequency linear array probe, configured to a frequency of 9–14 Hz.

The neonates were positioned in the supine, lateral, or prone position while in a quiet state. The lungs were divided into 6 regions per lung, for a total of 12 regions, using the bilateral nipples, posterior midline, posterior axillary line, anterior axillary line, and parasternal line as the dividing lines. The probe was scanned longitudinally perpendicular to the intercostal space and then gradually rotated 90° for transverse scanning. Static and dynamic ultrasonic images of each region were saved and recorded for subsequent image analysis. During the examination, personnel and instrument probes were disinfected and isolated.

The examiners received formal training in lung ultrasonography at the Beijing Obstetrics and Gynecology Hospital, Capital Medical University, which is the national training base for neonatal lung ultrasound. They were unaware of the basic clinical information of the neonates. The examining physicians jointly observed and interpreted the results.

### 2.4 Statistical analysis

The data for this study were analyzed using SPSS 20.0. Continuous data conforming to a normal or near-normal distribution were expressed as mean ± standard deviation (*x* ± *s*), and count data were expressed as cases (rates). The inter-group analysis was performed using *χ*^2^ test or Fisher's exact probability test for comparison between groups. Multivariate analysis was performed using logistic regression analysis, and the test level was *α* = 0.05. The level of statistical significance was set at *P* < 0.05.

## 3 Results

### 3.1 Participant demographics and clinical characteristics

There were 200 patients without pulmonary lesions at admission (average gestational age, 38.9 ± 1.8 weeks; 105 male and 95 female neonates; birth weight, 1,900–4,160 g). Moreover, there were 200 neonates with TTN (average gestational age, 37.7 ± 1.3 weeks; 124 male and 76 female neonates; birth weight, 1,700–4,820 g), including 132 with mild condition and 68 with severe condition, among which 60 patients received CPAP support, and 8 underwent tracheal intubation.

### 3.2 Comparison of each pulmonary ultrasonographic sign in each group

In the acute stage (within 6 h after birth), the pleural line was abnormal, and the A-line disappeared or decreased in all patients with TTN (Table 1). The differences in all ultrasonographic signs were significant when compared with the control group (*P* < 0.05). Moreover, there was no significant difference in these two signs based on the degree of TTN. In the acute stage, 91.2% of the patients with severe wet lungs had a white lung/dense B-line, and this incidence was significantly higher than that noted in neonates with mild wet lungs (3.8%, *P* < 0.05).

**Table 1:** Comparison of the ultrasonographic signs in the acute stage of mild and severe TTN (*) and between the TTN and control groups (#).

Ultrasonographic sign	TTN group #		Control group # (*n* = 200)	*P*-value*#
	Mild * (*n* = 132)	Severe * (*n* = 68)		
White lung/dense B-line	5 (3.8%)	62 (91.2%)	0 (0%)	<0.05*^#^
AIS	127 (96.2%)	6 (8.8%)	0 (0%)	<0.05*^#^
Abnormal pleural line, disappearance or decrease of A-line	132 (100%)	68 (100%)	0 (0%)	>0.05*
				<0.05^#^
Double lung points	84 (63.6%)	6 (8.8%)	0 (0%)	<0.05*^#^
Fused B-line	0 (0%)	0 (0%)	21 (10.5%)	>0.05*
				<0.05^#^
Pleural effusion	14 (10.6%)	8 (11.7%)	0 (0%)	>0.05*
				<0.05^#^

TTN, transient tachypnea of the newborn, transient respiratory distress and hypoxemia in neonates; AIS, alveolar-interstitial syndrome, a condition in which the fluid in the alveoli and pulmonary interstitium increases under pathological conditions, and multiple B-lines are presented on lung ultrasound.

### 3.3 Analysis of the neonates’ recovery time and its influencing factors

#### 3.3.1 Clinical recovery duration and complications

The clinical recovery durations for the 200 neonates were distributed as follows: 60.5% (121/200) recovered in 1–2 days, 35.0% (70/200) recovered in 3–4 days, and 4.5% (9/200) recovered in ≥5 days. The average clinical recovery durations of the neonates with mild illness, those that required non-invasive ventilation, and those that required invasive ventilation were 1.42 ± 0.50, 3.36 ± 0.51, and 6.00 ± 1.12 days, respectively. The differences between the groups were significant (*P* < 0.05). Among the 200 neonates, 40 had acidosis, 6 had pulmonary hypertension of the newborn (PPHN), 11 had ARDS, 7 had type II respiratory failure, 24 had patent ductus arteriosus (PDA), 22 had pleural effusion, 4 had heart failure, and 1 had pneumothorax.

#### 3.3.2 Univariate analysis of the clinical recovery duration

Groups A (recovery time <5 days) and B (recovery time ≥5 days) showed no significant difference in the incidence of acidosis, pleural effusion, PDA, and pneumothorax among the patients (*P* > 0.05) (Table 2). The intergroup differences regarding the number of patients with type II respiratory failure, ARDS, persistent PPHN, and heart failure were significant (*P* < 0.05). Moreover, the mean gestational age of the neonates in group B was <37 weeks, and the difference in gestational age between groups A and B was significant (*P* < 0.05).

**Table 2:** Univariate analysis of the clinical recovery duration of patients with TTN [comparison of gestational age, body weight and complications between groups A (clinical recovery duration < 5 days) and group B (clinical recovery duration ≥ 5 days)].

Variable		TTN group (*n* = 200)		*P*-value
		Group A (<5 days) (*n* = 191)	Group B (≥5 days) (*n* = 9)	
Gestational age (weeks)		37.8 ± 1.4	36.2 ± 1.3	<0.05
Weight (g)		3,230 ± 568	3,149 ± 475	>0.05
Complications (cases)	Acidosis	38	2	>0.05
	Type II respiratory failure	4	3	<0.05
	ARDS	5	6	<0.05
	PPHN	2	4	<0.05
	Pleural effusion	20	2	>0.05
	PDA	24	1	>0.05
	Heart failure	1	3	<0.05
	Pneumothorax	1	0	>0.05

TTN, transient tachypnea of the newborn, transient respiratory distress and hypoxemia in neonates; Type II respiratory failiure, global respiratory insufficiency with elevated CO_2_ levels; ARDS, acute respiratory distress syndrome, a clinical syndrome characterized by refractory hypoxemia.; PPHN, persistent pulmonary hypertension of the newborn; PDA, patent ductus arteriosus.

#### 3.3.3 Multivariate analysis of the clinical recovery duration

The factors associated with the recovery time, based on the univariate analysis, included late preterm status (34 weeks ≤ gestational age <37 weeks), type II respiratory failure, ARDS, PPHN, and heart failure as independent variables. Clinical recovery duration ≥5 days was used as the dependent variable and included in the multivariate logistic regression analysis. The results showed that type II respiratory failure, ARDS, PPHN, and heart failure were independent risk factors affecting the treatment recovery time ≥5 days in patients with TTN (all *P* < 0.05) (Table 3). Therefore, complications with these four conditions prolonged the recovery time (positive correlation).

**Table 3:** Logistic multivariate analysis of the factors influencing the clinical recovery duration (≥5 days).

Severe wet lung	*B*	SE	Beta	*t*	Significance	Exp(*B*) 95% CI	
						Lower limit	Upper limit
Acidosis	0.030	0.025	0.059	1.213	0.227	−0.019	0.080
Type II respiratory failure	0.384	0.055	0.340	7.032	0.000	0.276	0.491
ARDS	0.332	0.059	0.365	5.644	0.000	0.216	0.448
PPHN	0.255	0.068	0.210	3.738	0.000	0.120	0.389
Pleural effusion	0.055	0.032	0.083	1.727	0.086	−0.008	0.117
Late preterm	0.050	0.029	0.091	1.736	0.084	−0.007	0.107
Heart failure	0.276	0.089	0.187	3.120	0.002	0.102	0.451
PDA	−0.022	0.031	−0.035	−0.727	0.468	−0.083	0.038
Pneumothorax	−0.018	0.140	0.006	0.128	0.898	−0.258	0.293

ARDS, acute respiratory distress syndrome; PPHN, persistent pulmonary hypertension of the newborn; PDA, patent ductus arteriosus; CI, confidence interval.

### 3.4 Receiver operating characteristic (ROC) curve analysis of the hospitalization time (≥5 days)

**Figure 2:**
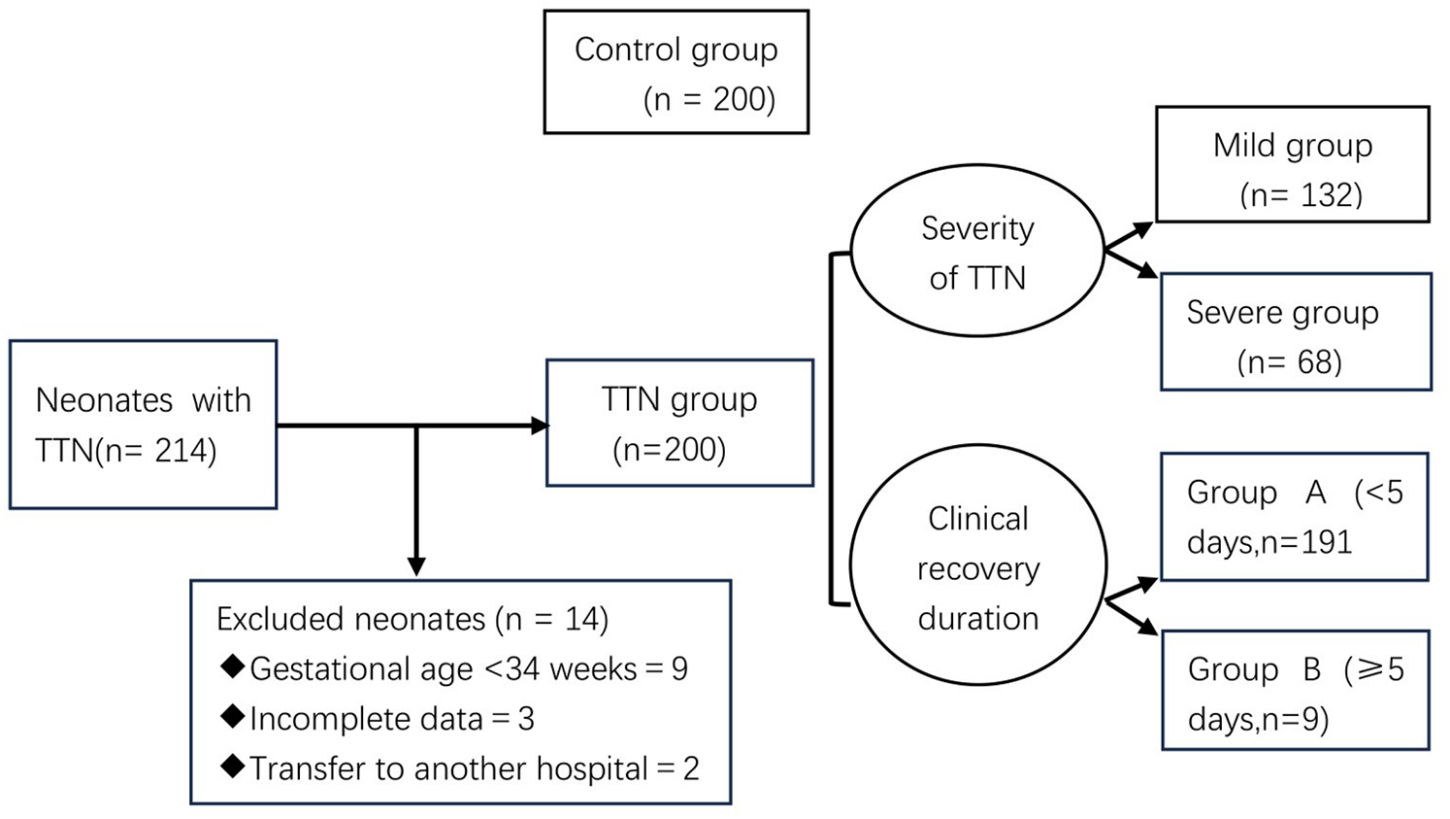
Receiver operating characteristic (ROC) curve analysis of hospitalization time.

An ROC curve was plotted to analyze the risk factors for the prolongation of the hospitalization time (≥5 days) in the neonates with TTN (Figure 2). The AUCs regarding complications with type II respiratory failure, ARDS, PPHN, and heart failure prolonging the hospitalization time (≥5 days) were 0.656, 0.820, 0.717, and 0.664, respectively. There was no significant difference between the risk factors. However, ARDS was associated with a significantly higher predictability (AUC: 0.820).

### 3.5 Typical cases in this study

#### 3.5.1 Case 1

**Figure 3:**
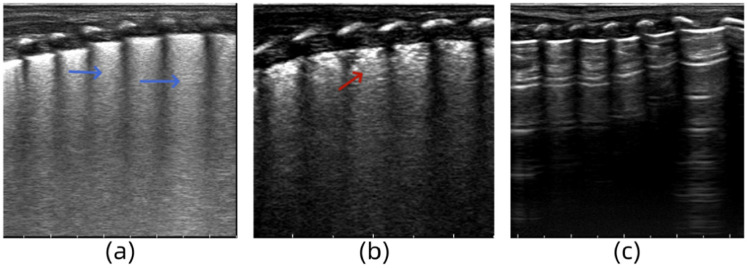
**(a)** Lung ultrasonography (LUS) reveals dense B-lines in lung fields. **(b)** LUS reveals a small number of B-lines in the intercostal spaces of the upper lung field and AIS manifestation in the lower lung field. **(c)** The LUS findings return to normal.

A female infant (G1P1) with a gestational age of 35 weeks was delivered via cesarean section. She was admitted to the hospital owing to tachypnea at birth, with a respiratory rate of 60 breaths/min and mild retraction. After admission, auscultation revealed coarse breath sounds in both lungs and moist rales. Chest radiography revealed thickened and blurred bilateral lung markings, and small patchy shadows. LUS revealed dense B-lines in both lung fields (Figure 3a). Arterial blood gas analysis revealed mild hypoxemia and nasal catheter oxygen supplementation was administered to maintain oxygen saturation. LUS re-examination at 24 h revealed a small number of B-lines in the intercostal spaces of the upper lung field and AIS manifestation in the lower lung field (Figure 3b). The LUS findings returned to normal following the 3-day re-examination (Figure 3c). Based on the clinical manifestations and auxiliary examination results, the infant was diagnosed with neonatal wet lungs.

#### 3.5.2 Case 2

**Figure 4:**
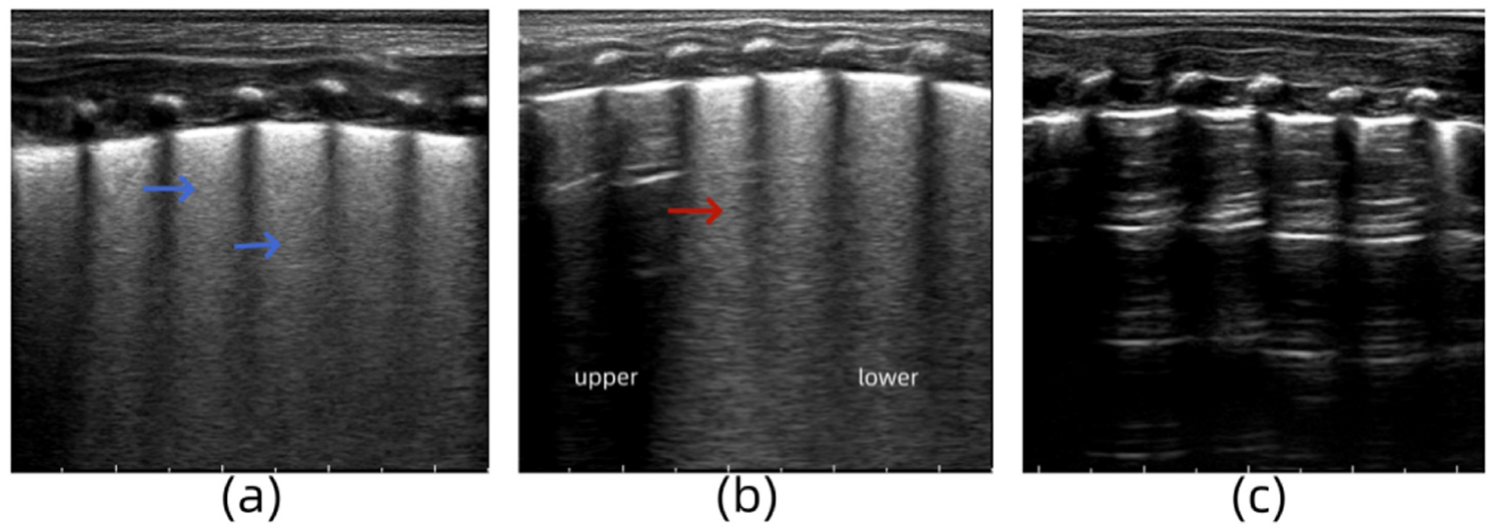
**(a)** Lung ultrasonography (LUS) examination reveals the disappearance of the pleural line and A-line in both lungs and a “white lung-like” change in both lung fields. **(b)** LUS re-examination shows “snowflake-like” bronchial inflation in the lungs. **(c)** LUS findings of both lungs return to normal.

A male infant (G1P1) with a gestational age of 36 weeks was delivered via cesarean section. He was admitted to the hospital with progressive dyspnea, obvious retractions, and cyanosis after birth. Arterial blood gas analysis revealed the following: pH, 7.329; partial pressure of carbon dioxide, 52.3 mmHg; and partial pressure of oxygen, 46.1 mmHg. LUS examination revealed the disappearance of the pleural line and A-line in both lungs and a “white lung-like” change in both lung fields (Figure 4a). The symptoms did not improve significantly under nasal noninvasive positive pressure ventilation. Tracheal intubation and mechanical ventilation were performed, and acidosis was corrected. Thirty-six hours after admission, the infant's symptoms did not improve, and LUS re-examination showed “snowflake-like” bronchial inflation in the lungs, which is a manifestation of ARDS (Figure 4b) (12). The ventilator parameters were adjusted, and bovine surfactant was instilled endotracheally. Seven days after admission, the infant's blood oxygen level was rechecked, and the LUS findings of both lungs returned to normal (Figure 4c). Based on the medical history, clinical manifestations, arterial blood gas analysis, and lung ultrasound examination, the infant was diagnosed with severe TTN complicated by ARDS.

#### 3.5.3 Case 3

**Figure 5:**
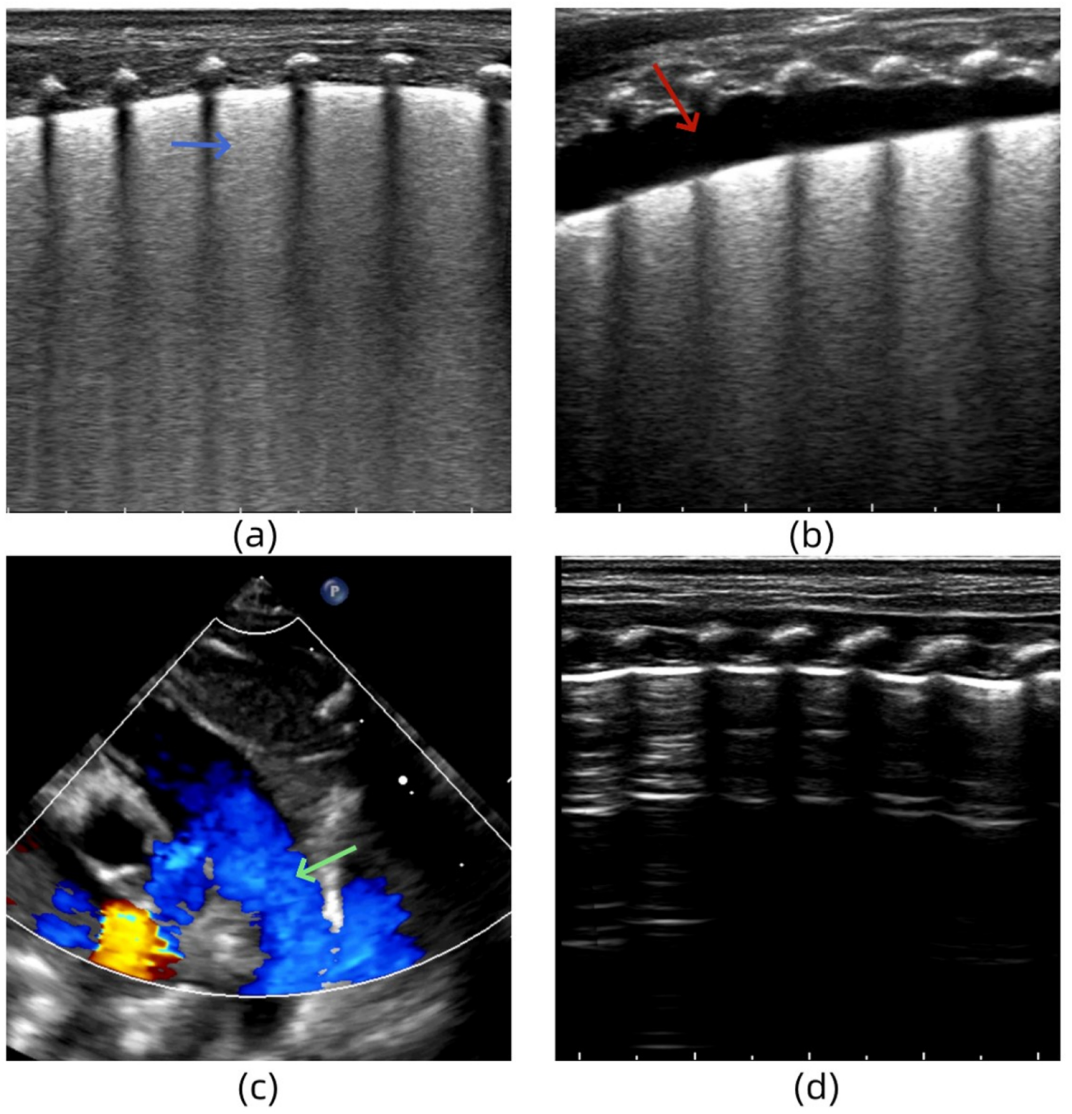
**(a)** Lung ultrasonography (LUS) reveals the dense B-lines in both lung fields. **(b)** LUS shows a small amount of pleural effusion in the lungs. **(c)** Echocardiography reveals a right-to-left shunt of the ductus arteriosus. **(d)** LUS findings return to normal.

A female infant (G2P1) with a gestational age of 33 weeks was delivered via cesarean section. She was admitted to the hospital for treatment because of obvious dyspnea, obvious retractions, and cyanosis of lips after birth. She received mechanical ventilation. After admission, LUS examination revealed an abnormal pleural line, disappearance of the A-line in both lungs and dense B-lines in both lung fields (Figure 5a). Two days after treatment, LUS re-examination showed a small amount of pleural effusion in the lungs (Figure 5b). Moreover, echocardiography revealed a right-to-left shunt of the ductus arteriosus, suggesting pulmonary hypertension (Figure 5c). The clinical team administered timely vasodilator treatment to reduce the pulmonary artery pressure. Six days after admission, the clinical indicators and LUS findings returned to normal (Figure 5d). Echocardiography also showed no abnormal shunt. Based on the medical history, clinical manifestations, arterial blood gas analysis, and cardiac and lung ultrasound examinations, the infant was diagnosed with severe TTN complicated by PPHN.

## 4 Discussion

TTN is caused by a delay in the absorption and clearance of lung fluid. When there is an increased amount of fetal alveolar fluid in the late stage of pregnancy, inadequate lymphatic transport function, or insufficient compression of the fetus during delivery, fluid may accumulate in the lung interstitium or alveoli, triggering TTN.

The main lung ultrasonographic feature of TTN is the formation of a waterfall sign due to the extensive distribution of B-lines in both lungs and the disappearance of the A-line. The B-line reflects the increase and degree of difference in water content in the alveoli and lung interstitium. If this line is diffusely increased and densely arranged, similar to a waterfall, extensive alveolar or interstitial water-increasing lesions are indicated. Studies have shown that the specificity and sensitivity were 78% and 90%, respectively. Compared with other auxiliary examination methods, such as radiography, LUS has superior advantages in monitoring alveolar or interstitial edema with low sensitivity (13–15). Different degrees of pulmonary edema form the main basis for the ultrasonic diagnosis of TTN. In this study, we found that white lungs or dense B-lines were mainly observed in patients with severe wet lungs. Moreover, all mild wet lungs cases manifested as AIS (fusion B-lines were manifested in more than two consecutive intercostal spaces; however, the rib acoustic shadow was still clearly visible). Compared with mild wet lung cases, in severe wet lung cases, the B-lines are denser, the clinical condition is more severe, and the disease course is longer. Therefore, the density of B-lines on lung ultrasound may be an indicator of disease severity. Some authors believe that the lung ultrasound score can be used to evaluate the TTN condition and monitor its clinical evolution (16, 17). However, others believe that this system is not accurate enough to evaluate pulmonary diseases and, therefore, do not advocate its use (18–20).

In addition, we observed that some severe cases displayed a new sign on LUS—an inconsistent degree of edema between the upper and lower lung fields, known as the double-lung point sign—within 12–48 h after birth. This may be due to ultrasonographic changes during the recovery period of patients with severe wet lungs. In order to evaluate the recovery period of severe TTN and assess whether this sign can be used for the evaluation of acute wet lungs, further studies are needed. However, this is not a specific sign of TTN and cannot be used in the differential diagnosis of the condition (5). Pleural effusion is a common complication of various pulmonary diseases and is difficult to detect using chest radiography (18). This study found that 11.0% of the neonates with TTN had various degrees of pleural effusion.

Lung ultrasound is more informative than traditional radiography in neonates with TTN. The traditional view is that neonatal wet lungs is a self-limiting condition that generally resolves spontaneously within 24–72 h with oxygen therapy (via oxygen hood) and symptomatic treatment (17). This study found that the average recovery time for patients with mild TTN was 1.42 ± 0.50 days and that for patients with severe cases treated with CPAP and tracheal intubation was 3.36 ± 0.51 days and 6.00 ± 1.12 days, respectively. Complications with type II respiratory failure, PPHN, ARDS, and heart failure would require a longer treatment time, suggesting that clinicians need to be vigilant about the presence of secondary diseases, such as PPHN and ARDS, and adjust treatment measures in time to avoid prolonging the neonate's disease course, increasing the family burden and endangering the neonate's life.

Although most cases of TTN have a good prognosis, some severe cases may have serious complications. TTN accompanied by hypoxemia and acidosis can cause spasms of the small pulmonary blood vessels (resulting in a continuous increase in pulmonary artery pressure and right heart pressure) and a right-to-left shunt at the level of the foramen ovale and ductus arteriosus in the newborn, resulting in right heart failure, severe hypoxemia, and respiratory failure. Pulmonary diseases, such as TTN and ARDS, increase the risk of pneumothorax (21).

In one of the 200 TTN cases, the neonate suddenly experienced aggravated dyspnea, decreased partial pressure of oxygen in the blood, and collapsed lungs on chest radiography on the second day of hospitalization. The clinician quickly diagnosed neonatal pneumothorax and performed puncture and aspiration. The timely diagnosis and treatment reduced the risk of prolonging the recovery time of the neonate. The evolution of neonatal TTN and ARDS is similar, and TTN may occur secondary to ARDS. Before the implementation of LUS in the ward, it was sometimes difficult to distinguish it clinically, thereby increasing unnecessary treatments. With the assistance of high-resolution LUS, 11 cases of concurrent ARDS in the TTN group were detected in a timely manner, and the clinical team provided surfactant treatment to avoid the serious consequences of treatment delay.

All 200 neonates with TTN in this study were eventually cured and discharged without any deaths, indicating that TTN is not an invasive disease. However, our study has certain limitations. Owing to the small sample size, the study included only 68 patients with severe TTN. Future multicenter and large-sample studies are needed to validate our findings and determine the accuracy of the results. In addition, further follow-up of neonates with severe TTN is required to better assess their lung development.

In conclusion, LUS is a real-time, flexible, and non-invasive auxiliary examination tool that minimizes radiation exposure and transportation in neonates. It can be used for the initial screening, dynamic follow-up, and treatment evaluation of pulmonary diseases. It can also be used to evaluate the severity of TTN and assist in monitoring the clinical outcomes of neonatal wet lungs.

## Data Availability

The raw data supporting the conclusions of this article will be made available by the authors, without undue reservation.
